# Synchronous Primary Adenocarcinomas of the Hepatic and Splenic Flexures Presenting as Acute Large Bowel Obstruction in a 38-Year-Old Man: A Case Report

**DOI:** 10.7759/cureus.107024

**Published:** 2026-04-14

**Authors:** Jasneet Gill, Hunter W Brady, Chimezie Mbachi

**Affiliations:** 1 Department of Internal Medicine, Tennova North Knoxville Medical Center, Knoxville, USA; 2 Department of Medicine, Lincoln Memorial University DeBusk College of Osteopathic Medicine, Knoxville, USA; 3 Department of Gastroenterology, Tennova North Knoxville Medical Center, Knoxville, USA

**Keywords:** colon adenocarcinoma, large bowel obstruction, multifocal colon cancer, subtotal colectomy, synchronous colorectal cancer, young adult malignancy, young onset colorectal cancer

## Abstract

Synchronous primary colorectal cancers (CRCs) are uncommon and are more frequently identified in older patients or in association with hereditary cancer syndromes. Although the incidence of early-onset CRC (EOCRC) is increasing, synchronous primary tumors in young individuals without identifiable risk factors remain rare. Presentation as acute large bowel obstruction further complicates diagnosis because complete preoperative colonoscopic evaluation is often not feasible.

We report the case of a 38-year-old man with no significant medical or family history who presented with a two-day history of worsening generalized abdominal pain, nausea, vomiting, and constipation. Computed tomography (CT) demonstrated partial large bowel obstruction at the hepatic flexure. A water-soluble contrast enema subsequently revealed two distinct apple-core lesions at the splenic and hepatic flexures. The patient underwent emergent exploratory laparotomy with subtotal colectomy and ileocolic anastomosis. Intraoperatively, a 3.5 cm obstructing mass at the splenic flexure and a 4.5 cm polypoid mass at the hepatic flexure were identified.

Histopathologic examination confirmed moderately differentiated (grade 2) adenocarcinomas in both lesions with no lymphovascular or perineural invasion. Eighteen lymph nodes were examined, and all were negative for metastasis. Surgical margins were negative. Immunohistochemistry showed intact mismatch repair protein expression (microsatellite stable). Preoperative and postoperative carcinoembryonic antigen levels were normal. Both tumors were staged as pT3N0M0 (stage IIA). The postoperative course was complicated by small bowel obstruction that resolved with conservative management; the patient was discharged after a seven-day hospitalization and remained well at the three-month follow-up with planned surveillance.

This case highlights the importance of considering synchronous colorectal malignancies even in younger patients without traditional risk factors. When a complete colonoscopic assessment is not possible due to obstruction, adjunctive imaging such as contrast enema and extended resection are essential for adequate oncologic management.

## Introduction

Colorectal cancer (CRC) is the third most commonly diagnosed malignancy worldwide and remains a leading cause of cancer-related mortality, with more than 1.9 million new cases and over 900,000 deaths annually [1]. Although historically considered a disease of older adults, recent epidemiologic data demonstrate a concerning rise in early-onset CRC (EOCRC) among individuals younger than 50 years, many of whom lack traditional risk factors [2]. Most CRC cases (approximately 92% to 97%) present as solitary primary tumors [3].

Synchronous CRCs are defined as two or more distinct primary colorectal tumors identified simultaneously or within six months of the initial diagnosis. These tumors must represent independent primary neoplasms rather than local extension or metastatic spread, distinguishing them from metachronous tumors, which develop more than six months after the index malignancy [4]. Synchronous tumors account for approximately 3% to 5% of CRC cases and occur more frequently in older patients [5]. They are commonly associated with hereditary cancer syndromes, including Lynch syndrome and familial adenomatous polyposis, as well as microsatellite instability and other molecular alterations that predispose to multifocal tumorigenesis [5].

Accurate identification of synchronous lesions is essential because their presence influences staging, operative planning, and long-term surveillance. Complete preoperative colonoscopic evaluation is recommended to exclude additional lesions; however, this may not be feasible in patients presenting with acute large bowel obstruction [6]. In such cases, incomplete visualization increases the risk of missed synchronous tumors, potentially resulting in inadequate resection or the need for subsequent surgery.

We report a rare case of synchronous primary colonic adenocarcinomas at the hepatic and splenic flexures causing acute large bowel obstruction in a 38-year-old man without identifiable genetic or environmental risk factors. This presentation highlights a diagnostic challenge uncommon in EOCRC: the need for adjunctive imaging (contrast enema) to detect multifocal disease preoperatively and the rationale for extended resection when lesions involve separate vascular territories.

## Case presentation

A 38-year-old man presented to the emergency department with a two-day history of worsening generalized abdominal pain accompanied by nausea, vomiting, and constipation. He reported intermittent constipation for the preceding two months but denied weight loss, hematochezia, melena, or changes in appetite. He had no significant past medical history or family history of CRC or hereditary cancer syndromes. His body mass index was normal (22.5 kg/m²); he took no regular medications, and he had no comorbidities.

On examination, the patient was mildly febrile and tachycardic. Abdominal examination revealed diffuse tenderness without guarding or rebound tenderness. Laboratory studies showed leukocytosis (white blood cell count 14,000/μL; reference range 4,000-11,000/μL), mild anemia (hemoglobin 9.8 g/dL; reference range 13.5-17.5 g/dL), and a mildly elevated serum lactate (2.1 mmol/L; reference range 0.5-2.2 mmol/L).

**Figure 1:**
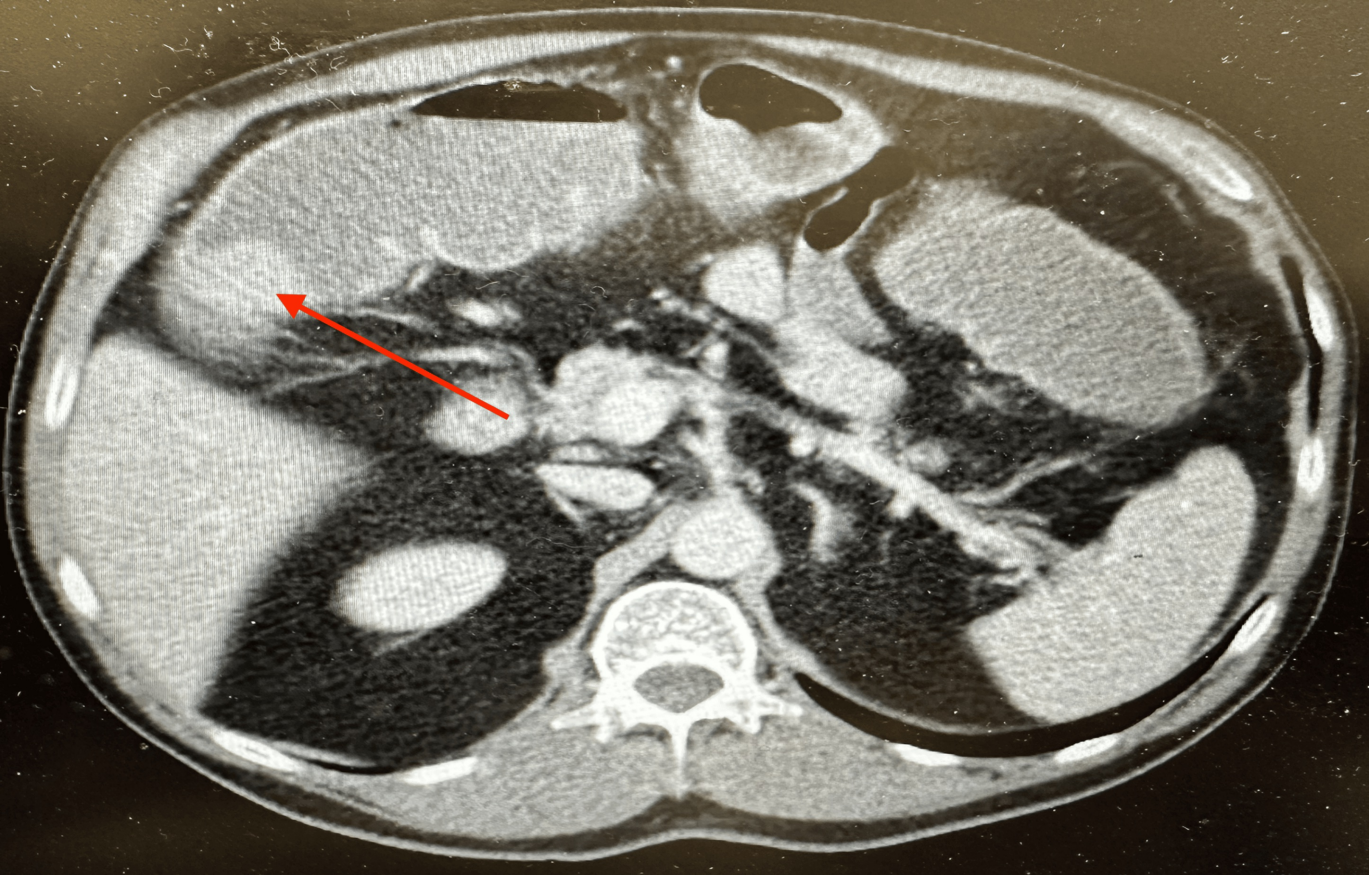
Contrast-enhanced computed tomography (CT) of the abdomen demonstrating circumferential wall thickening at the hepatic flexure. Axial CT image of the abdomen with intravenous contrast showing focal circumferential colonic wall thickening at the hepatic flexure (red arrow), resulting in luminal narrowing and proximal colonic dilation consistent with partial large bowel obstruction.

Contrast-enhanced computed tomography (CT) of the abdomen and pelvis demonstrated focal circumferential wall thickening at the hepatic flexure with luminal narrowing and proximal colonic dilation, consistent with partial large bowel obstruction (Figure 1). A subsequent water-soluble contrast enema revealed apple-core lesions at both the splenic flexure and hepatic flexure, raising concern for synchronous obstructing malignancies. Preoperative colonoscopy was not performed because of the acute obstructive presentation.

The patient underwent emergent open laparotomy. Intraoperatively, a firm 3.5-cm obstructing mass was identified at the splenic flexure and a 4.5-cm polypoid mass at the hepatic flexure. A subtotal colectomy with ileocolic anastomosis was performed to address tumors in separate vascular territories while ensuring adequate oncologic margins.

Microscopic examination of both lesions revealed a moderately differentiated (grade 2) adenocarcinoma characterized by gland formation and nuclear atypia. Unfortunately, the original histopathology slides, which were located at an outside institution, were unavailable for review despite multiple attempts to obtain them. There was no perineural or lymphovascular invasion. A total of 18 lymph nodes were examined, all of which were negative for metastatic disease. Surgical margins were negative. Immunohistochemistry showed intact expression of MLH1, MSH2, MSH6, and PMS2, consistent with microsatellite-stable status. Preoperative and postoperative carcinoembryonic antigen levels were within normal limits. Both tumors were staged as pT3N0M0 (pathologic stage IIA).

The postoperative course was complicated by a small bowel obstruction that resolved with conservative management. Postoperative chest CT demonstrated small indeterminate nodular opacities in the right lung base, with plans for interval radiographic surveillance. The patient was scheduled for a follow-up colonoscopy and repeat imaging three months postoperatively. Given his young age at diagnosis, genetic counseling and germline testing were recommended.

## Discussion

Synchronous primary CRCs are uncommon but clinically significant, particularly in patients younger than 45 years without identifiable risk factors. Although they are more often described in older adults and in association with hereditary cancer syndromes, this case highlights an atypical presentation defined by early-onset disease, the absence of known risk factors, and acute large bowel obstruction [7,8]. As such, colorectal malignancy should remain in the differential diagnosis for younger patients presenting with bowel obstruction, even in the absence of classic warning signs or a contributory family history.

At 38 years of age, this patient falls within the EOCRC population, a group in which incidence has risen steadily over the past two decades [9]. EOCRC is also more likely to present at an advanced stage and may demonstrate more aggressive clinicopathologic features than CRC diagnosed in older adults [10]. Even so, synchronous primary tumors remain relatively uncommon in this age group [5]. The presence of two pT3N0M0 adenocarcinomas in a young patient without an established predisposition underscores the evolving epidemiology of CRC and suggests that multifocal tumorigenesis may occur even in the absence of a recognized hereditary syndrome.

Although no hereditary syndrome was identified on initial evaluation, the patient’s age and multifocal presentation warranted formal germline testing [11]. Evaluation for mismatch repair deficiency or microsatellite instability is particularly important in this setting, as these abnormalities are associated with synchronous or multifocal disease and may carry both prognostic and therapeutic implications [12]. This case therefore reinforces the need for a careful hereditary cancer assessment even when family history is negative, as de novo mutations, incomplete penetrance, or unrecognized familial syndromes may underlie such presentations.

This case also illustrates the diagnostic limitations imposed by obstructing CRC. Complete preoperative colonoscopy is the standard approach for excluding synchronous lesions [13]; however, full colonic evaluation is often not feasible in the setting of acute obstruction. In this patient, contrast enema was not simply confirmatory but management-altering, as it allowed preoperative identification of both tumors. Without adjunctive imaging, the second lesion might have been missed, potentially resulting in incomplete oncologic resection or the need for subsequent reoperation.

Subtotal colectomy with ileocolic anastomosis was an appropriate operative strategy given the presence of tumors in separate vascular territories at the splenic and hepatic flexures. In synchronous CRC, surgical planning must balance oncologic adequacy with preservation of postoperative bowel function. When lesions are distributed across distinct colonic segments, a more extensive resection may be necessary to ensure adequate margins, remove all gross disease, and reduce the risk of leaving behind occult synchronous pathology.

The postoperative small bowel obstruction resolved with conservative management. The indeterminate pulmonary nodules identified postoperatively require interval surveillance to exclude metastatic disease. Given the patient’s young age and multifocal presentation, close long-term follow-up is warranted. Current guidelines recommend surveillance colonoscopy within one year of resection [14].

## Conclusions

This case demonstrates that synchronous primary colonic adenocarcinomas may present as acute large bowel obstruction even in young adults without traditional colorectal cancer risk factors. Clinicians should maintain a high index of suspicion for multifocal disease in younger patients with obstructive symptoms and pursue evaluation of the entire colon whenever possible. In emergent settings where complete colonoscopic assessment is not feasible, adjunctive imaging (such as water-soluble contrast enema) is critical for detecting additional lesions and guiding surgical strategy. When synchronous tumors involve separate vascular territories, subtotal colectomy should be considered to ensure adequate oncologic resection. Increased awareness of such atypical presentations, combined with routine molecular testing, may improve diagnostic accuracy, surgical planning, and long-term outcomes.
